# The risk of contact between visitors and *Borrelia burgdorferi*-infected ticks is associated with fine-scale landscape features in a southeastern Canadian nature park

**DOI:** 10.1186/s12889-024-18673-w

**Published:** 2024-04-26

**Authors:** Ariane Dumas, Catherine Bouchard, Pierre Drapeau, L. Robbin Lindsay, Nicholas H. Ogden, Patrick A. Leighton

**Affiliations:** 1 Department of Pathology and Microbiology, Faculty of Veterinary Medicine, Université de Montréal, Saint-Hyacinthe, QC Canada; 2 Epidemiology of Zoonoses and Public Health Research Unit (GREZOSP), Faculty of Veterinary Medicine, Université de Montréal, Saint-Hyacinthe, QC Canada; 3 Public Health Risk Sciences Division, National Microbiology Laboratory, Public Health Agency of Canada, Saint-Hyacinthe, QC Canada; 4 Department of Biological Sciences, Centre for Forest Research, Université du Québec À Montréal, Montreal, QC Canada; 5 One Health Division, National Microbiology Laboratory, Public Health Agency of Canada, Winnipeg, MB Canada

**Keywords:** Local scale, Risk assessment, Tick-borne disease, Public natural areas, Trail network, Spatial analysis, Epidemiology

## Abstract

**Background:**

Infectious diseases are emerging across temperate regions of the world, and, for some, links have been made between landscapes and emergence dynamics. For tick-borne diseases, public parks may be important exposure sites for people living in urbanized areas of North America and Europe. In most cases, we know more about the ecological processes that determine the hazard posed by ticks as disease vectors than we do about how human population exposure varies in urban natural parks.

**Methods:**

In this study, infrared counters were used to monitor visitor use of a public natural park in southern Quebec, Canada. A risk index representing the probability of encounters between humans and infected vectors was constructed. This was done by combining the intensity of visitor trail use and the density of infected nymphs obtained from field surveillance. Patterns of risk were examined using spatial cluster analysis. Digital forest data and park infrastructure data were then integrated using spatially explicit models to test whether encounter risk levels and its components vary with forest fragmentation indicators and proximity to park infrastructure.

**Results:**

Results suggest that, even at a very fine scales, certain landscape features and infrastructure can be predictors of risk levels. Both visitors and *Borrelia burgdorferi*-infected ticks concentrated in areas where forest cover was dominant, so there was a positive association between forest cover and the risk index. However, there were no associations between indicators of forest fragmentation and risk levels. Some high-risk clusters contributed disproportionately to the risk distribution in the park relative to their size. There were also two high-risk periods, one in early summer coinciding with peak nymphal activity, and one in early fall when park visitation was highest.

**Conclusions:**

Here, we demonstrate the importance of integrating indicators of human behaviour visitation with tick distribution data to characterize risk patterns for tick-borne diseases in public natural areas. Indeed, understanding the environmental determinants of human-tick interactions will allow organisations to deploy more effective risk reduction interventions targeted at key locations and times, and improve the management of public health risks associated with tick-borne diseases in public spaces.

**Supplementary Information:**

The online version contains supplementary material available at 10.1186/s12889-024-18673-w.

## Introduction

Shifts in the geographic distributions of infectious diseases are currently being observed in transforming ecosystems, highlighting the complex and dynamic interface between landscapes and disease ecology [1, 2]. For example, tick-borne diseases have been expanding for several years in many parts of the temperate world. In Europe and North America, the distribution and abundance of ixodid ticks and their reservoir hosts has been linked to land use change and climate change [3–5]. At the same time, human behavior risk factors are leading to increased contact with ticks [6–8] and as a result, a larger portion of the human population is being exposed to ticks. This is causing an increase in tick-borne disease incidence rates in many regions of the world [9–12]. While epidemiologists have emphasized the importance of incorporating landscape characteristics into studies of the ecological dynamics of infectious disease emergence, less attention has been directed to the human factors modulating the risk of being exposed to vectors and eventually developing disease [1].

To effectively manage the risk associated with diseases in different regions and landscapes, it must first be accurately defined, assessed, and the factors that determine it understood [13]. Risk represents the likelihood that an adverse event will occur, given the consequences it would cause. These consequences depend on the vulnerability of the population of interest to the hazard and are determined by factors such as its level of exposure and coping capacity [13–15]. In the context of tick-borne diseases, the hazard level at a given location or time is typically represented by the number of pathogen-infected ticks present (hereafter referred as the “tick hazard”; [16]). The density of these ticks in the environment depends on a set of ecological conditions that allow them, their hosts, and the pathogens that circulate between them to complete their life cycles [17, 18]. Exposure represents the degree to which humans encounter vectors. It is related to land use, accessibility and attractiveness of places where ticks are present [19]. The consequences of exposure to the hazard are ultimately modulated by a range of social and behavioral factors that determine the coping capacity of the population [15]. For example, the use of tick repellents decreases the likelihood that an individual will be bitten by a tick, and tick checking decreases the likelihood that an individual will become infected with a pathogen if bitten by a tick. The degree of awareness and adoption of these personal protective measures in populations may vary according to socioeconomic factors and regional endemicity [20]. Overall, all these elements − hazard, exposure and coping capacity − and the way they interact are likely to vary across landscapes and populations [15, 21].

Several studies have examined the relationship between the level of forest fragmentation in landscapes and the risk of tick-borne diseases to the human populations present. In North America, this research has been conducted primarily in residential agroforestry landscapes [16]. Several of these studies have shown that the risk of tick-borne diseases is generally higher in areas where the forest is fragmented than in more homogeneous areas, such as large forest stands or urban areas [5, 22]. Different mechanisms have been suggested to explain this association. One is that the good adaptation of tick hosts (wildlife species used by ticks for reproduction or serving as reservoirs for pathogen transmission) to the varied habitats present in fragmented landscapes and their concentration in small fragments could enhance enzootic transmission. In addition, the increased presence of areas such as forest-field transition zones (ecotones) are favorable for contacts between human and infected ticks [3].

In urban areas, the risk is generally concentrated in smaller portions of the territory, such as publicly accessible green spaces and natural conservation parks [23, 24]. However, we do not know currently whether similar processes linking forest fragmentation to increased tick-borne disease risk also take place in these environments. Indeed, in public nature parks, the causes of forest fragmentation, its general importance, and the way it impacts ticks, wildlife and people distribution and thus the tick-borne diseases risk may be different. The natural or anthropogenic presence of different types of habitats such as herbaceous or shrubby areas, the presence of road and trail networks, or built features (e.g., service buildings, lookouts) are all elements that can lead to forest fragmentation in the context of natural parks [25]. These elements could influence the distribution of tick hazard, human exposure, or both. For example, trail networks are generally the principal driver of visitors’ spatial distribution across the different areas of a park [26–28]. Trails can also create edge effects, causing changes in the adjacent vegetation, altering abiotic conditions such as light and affecting wildlife and tick presence [29, 30]. The presence of features like viewpoints, waterbodies or facilities like picnic areas and playgrounds can influence landscape attractiveness for people [28, 31, 32] and therefore influence the level of the park users’ exposure.

Limited research has been conducted to characterize the risk associated with tick-borne diseases in the context of public parks of North America. In parks of southern Quebec (Canada), Ripoche et al. [30] found more nymphs in forest habitat adjacent to park trails (measured at points between 20 and 60 m from trails) than directly along the trail edges and higher nymph densities near trails with soil surfaces compared to those with gravel surfaces. Hotspots of high nymph densities were observed in less frequented parts of the parks, while cold spots were located in high-traffic areas such as park entries, trailheads and at park edges, close to residential neighborhoods. Falco and Fish [33] found lower distances to encountering nymphal or adult *I. scapularis* in plots that were randomly sampled throughout parks than in areas of high public use identified by park managers, suggesting that high-use areas were characterized by lower tick densities. In view of these results, the authors proposed that a high human presence could limit the local abundance of tick hosts. Indeed, animals generally respond to human presence in a manner similar to their response to predation, i.e., by avoiding or underutilizing highly disturbed areas [34]. If fewer hosts are available locally, the probability for ticks to complete their life cycle may be reduced, eventually limiting their local abundance. Through these mechanisms, the risk of tick-borne disease transmission could be influenced by the intensity of human presence in public natural areas. However, in these two studies, space use by people and hosts were not directly measured. Overall, quantitative relationships between the tick hazard and population exposure and fine-scale habitat characteristics in parks have not yet been clearly established. In highly used urban natural areas, improving this baseline knowledge would be particularly relevant to inform local tick-borne diseases risk reduction efforts, for both public health and park managers.

Here, we present a case study of the spatial and temporal variation in risk across a periurban nature park with an emerging risk for Lyme disease (LD) transmission. To do so, we integrate population exposure and tick hazard data. These are respectively represented by each trail’s usage intensity and the density of infected nymphs (DIN) with the LD agent, *Borrelia burgdorferi*, in the vicinity of the trails. This allows us to create an indicator of the probability of human-tick contact (risk) across the park. We also verify the presence of risk hotspots. From a park risk management perspective, the deployment of risk reduction interventions in these hotspot areas could have a significant positive impact. We explore which features of the park landscape are associated with risk and its two components, hazard and exposure. We hypothesize that forest fragmentation is a determinant of risk distribution across the park. In this park context, the level of forest fragmentation can be represented by indicators such as trail density and the presence of developed areas. First, these elements could generate ecological transition zones (ecotones) between two types of habitats, i.e. zones where tick-host reservoir interactions favour the transmission of pathogens and therefore an increase in the tick hazard. Secondly, a high density of trails and the presence of developed elements could promote the attractiveness and accessibility of the sectors, parameters associated with high visitor traffic and therefore an increase in exposure. Our results will allow for better management of emerging tick-borne diseases in nature parks and contribute to the body of knowledge on the links between fine-scale landscape ecology and the dynamics of tick-borne diseases.

## Methods

### Study site

**Fig. 1:**
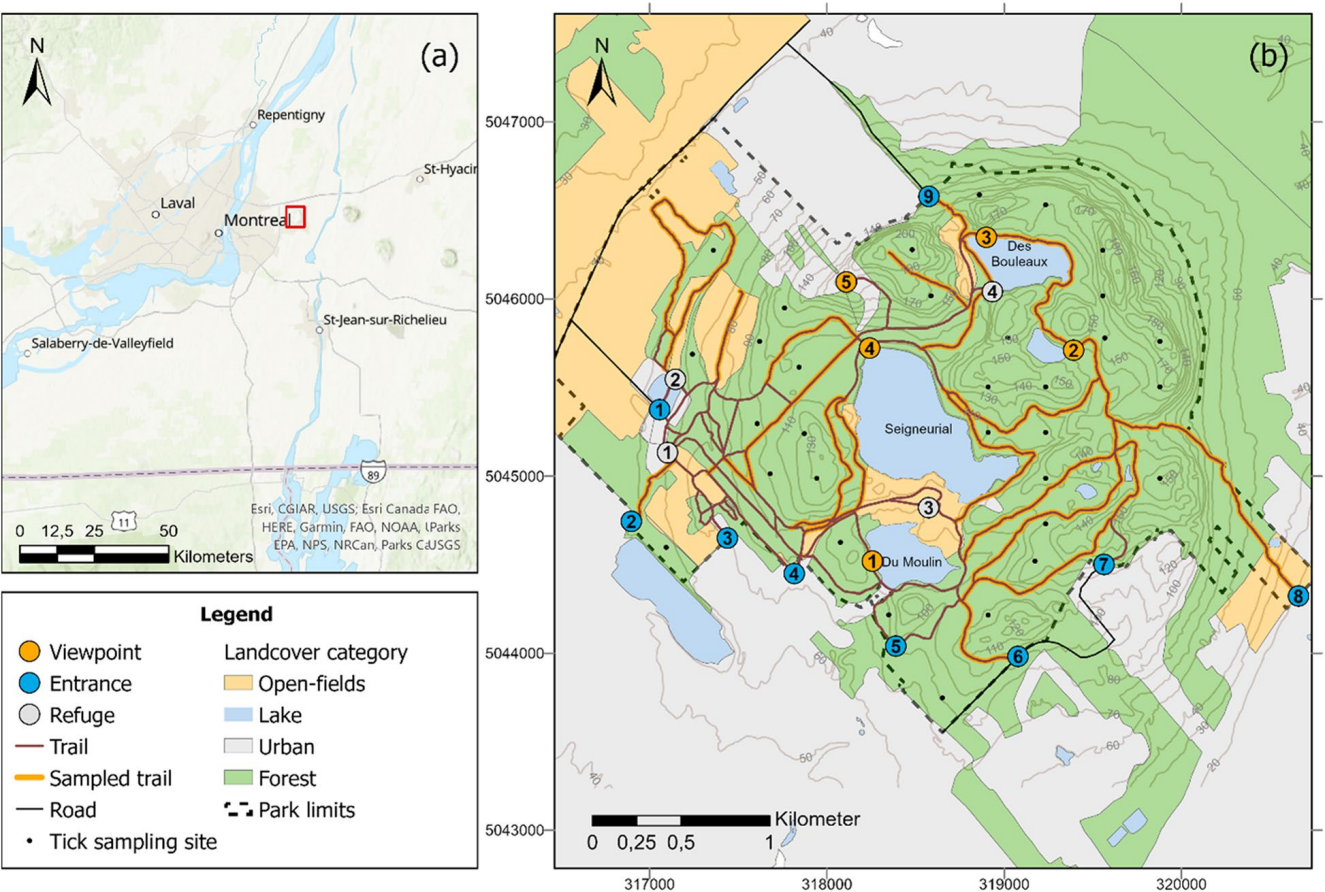
The location of the study site, Mont-Saint-Bruno National Park, in relation to the city of Montreal, Quebec, Canada (**a**). The location of the sampling sites, landcover types and infrastructures within the park, designated by the identification numbers in the circles (**b**)

The study took place in Mont Saint-Bruno National Park, located in southern Quebec, Canada. Mount Saint-Bruno (Fig. 1), with an altitude of 218 m, is dominated by stands of deciduous forest (mainly sugar maple (*Acer saccharum*), American beech (*Fagus grandifolia*) and red oak (*Quercus rubra*)). Humans have inhabited and transformed this forest over the course of history, and the forest is fragmented by private properties, landscaped gardens, former orchards and open areas where herbaceous and bushy vegetation dominate. Located just outside of the city of Montréal, this small park of less than 10 km^2^ is a popular destination for hikers and attracts approximately one million visitors each year. The park's 27 km unpaved trail network includes wide trails where cyclists and authorized vehicles can circulate, and narrow trails reserved for pedestrians. Several facilities are available to visitors, including a visitor center, shelters, picnic areas, a playground and several lookouts. Sampling conducted in the park as part of the provincial tick surveillance program detected the presence *I. scapularis* in 2007 and the first *B. burgdorferi* infected ticks in 2012, with tick density increasing steadily in the park ever since.

### Tick sampling and diagnostic testing

Thirty-two sampling sites were systematically located in tick habitat. They were spaced at 325 m intervals throughout the forested zones of the park using a grid-based sampling design. Some sites were excluded if they were predominantly wetlands or open fields, had major access barriers (such as a cliff or very steep terrain), or were crossed by trails (Fig. 1). At these 32 forested sites, ticks were collected in 2017 and 2018.1 m^2^ flannel cloth was dragged across the forest floor along 260 m transects, once a month from May to October when ticks are most active. Sampling was conducted between 8 am and 4 pm on days without rain. The investigators walked at a natural and steady pace while ensuring the flannel dragged the ground correctly and stopping at every 25 m interval along the transect to check the flannel and count and collect any attached ticks. Ticks were preserved in 1.5 ml vials containing 70% ethanol.

Tick species identification was confirmed at the National Microbiology Laboratory (NML) in Winnipeg, Canada using taxonomic keys [35–37]. Then a subset of up to 30 nymphs per location per year were tested using RT-PCR to detect *B. burgdorferi* infection*.* Extraction of tick DNA was performed according to the manufacturer’s protocol using QIAGEN®DNeasy®96 Tissue kits (QIAGEN Inc., Mississauga, ON, Canada). Extracted DNA was screened using a duplex real-time PCR assay targeting the *23S* gene of *Borrelia spp.* [38]. *Borrelia*-positive samples were subsequently tested for *B. burgdorferi* sensu stricto using a second *ospA*/*flab* duplex assay [39].

For each site-year, the density of nymphs (DON) was calculated as the total number of nymphs collected per 100 m^2^ over the entire tick sampling season. The annual park-level nymphal infection prevalence (NIP) was estimated as the proportion of *B. burgdorferi* positive nymphs among those tested. Lastly, the density of infected nymphs (DIN) at each site-year was estimated by calculating site-year DON*yearly NIP.

### Monitoring trail use and park visitation

Passive infrared counters (3 model *TRAFx trail counters*, *TRAFx™*, maximum range 6 m; 6 model *VigilMeter, Vigil™*, maximum range 4.5 m) were deployed at 20 trail segments (the portion of trail between two intersections) in 2017 and 10 additional segments in 2018. On a rotating basis, each trail segment received a counter for a period of one to two weeks, during the period from May to October. The counters were placed within 4.5 m of the opposite edge of the trail and recorded the number of passes per hour. To test the agreement of the data collected by the two different types of counters, we positioned one of each type at the same location (control trail) for a period of two days and compared the data collected by each with a Pearson correlation test. Since the correlation was very high (0.99, *p* < 0.001, *n* = 30) it was considered that the detection sensitivity of the two types of counters was comparable.

To characterize the type of detection recorded by the counters, infrared cameras *(Spypoint™*, model *Iron10*) were also deployed on every trail segment sampled in 2017. The cameras were deployed on a tree, within 2 m of the counter and pointed in the same direction. The cameras were programmed to take a single picture per detection, with a maximum of one picture per minute. For each picture, an observer manually counted the number of adults, children, vehicles and animals detected. These data were then used to adjust the counters detection data. First, the data were adjusted to consider only pedestrians and cyclists, which is the population at risk in the study. Therefore, using the camera data, the proportion of all pictures that detected people in each trail segment was calculated. Second, the number of individuals per counter detection was adjusted using the average size of groups of people on the pictures taken in each segment.

The number of people visiting the park varies across seasons and days of the week. Therefore, the data collected by the trail counters was also adjusted to make them comparable, regardless of the days sampled at each location. This was accomplished using the number of daily passes sold and an estimate of attendance by annual pass holders generated by the park managers [40]. From this, a baseline daily value of the approximate total visitors to the park on each day was calculated. Then the adjusted count values at each trail segment were divided by the corresponding baseline daily values. This adjustment methodology relies on the assumption that the distribution pattern of visitors throughout the park was stable over the study period. Indeed, the distribution of people in public natural areas is determined by infrastructure and landscape characteristics [26–28, 31, 32] and no modifications were made in or around the park during the study period that might have affected these features and hence the spatial distribution of visitors. Daily adjusted visitor counts were then averaged over the period of sampling at each trail segment. Finally, these values were interpolated to unsampled locations using segment-based ordinary kriging, a geostatistical interpolation technique that considers the degree of spatial variation of the data as a function of their topological distance in a network [41]. The model was implemented in R using the package *SK*, version 1.1 [42]. The final output of these steps was a standardized trail use index for the entire park trail network, which was then used as an exposure index in subsequent analyses.

This index of trail use by visitors does not distinguish between counts at the individual level. That is, this index could be the result of some individuals being counted while passing through the same trail segment multiple times, and other individuals being counted only once while hiking a circular route. This is not expected to be a major problem because the trails all allow for circular routes, and because the index is designed to assess exposure at the population level. In fact, at the population level, exposure is a function of population size, exposure time, and other behavioral factors that are beyond the scope of this study [15]. Therefore, in the remainder of the text, any reference to the number of visitors will implicitly include both types of detections (same or multiple individuals).

### Spatial analysis of risk levels

Relative risk indices were estimated for each trail segment across the park. Here, the risk was conceptualized as a probability of contact between people and infected ticks, where the highest density of infected nymphs (tick hazard), together with the highest concentration of people (population density, as a proxy for exposure) in some locations and/or time points represent the riskiest situations. The rationale for this is that if aggregates of high-risk are detected in a geographic and/or temporal space, these spaces could be targeted to develop interventions to prevent tick bites for the greatest number of people and potentially reduce LD cases. For this, tick hazard indices were first estimated. Site-year DIN values were used, which were interpolated across the park. Empirical variograms were fit using the package *automap* version 1.0.14 [43] in R and the resulting parameters were then used in interpolation kriging models implemented in the package *sp*, version 1.4.5 [44]. Then, the average values at each trail’s buffered polygon (circle-shaped 25 m buffer) were extracted to obtain the trail segment-based tick hazard indices. Next, population exposure in each year was estimated (i.e. an estimate of the number of people who have used each trail segment), by cross-producing the total number of registered visitors to the park in each year with the relative trail use index obtained in previous steps. Finally, the relative risk indices were calculated by multiplying the tick hazard and population exposure indices, in both years of the study.

Then these risk indices were used to perform spatial cluster analysis for each year of the study. This analysis was performed to detect potential risk hotspots, which could then be targeted for risk management. The unit of analysis was each trail segment of the park and its immediate surroundings, represented by a 25 m buffer around the trails. For this analysis the Local Moran’s I statistics were computed [45] using the Cluster and Outlier Analysis tool in ArcGIS Pro version 2.8.0. 999 permutations were run to assess the significance of the patterns, polygons were considered as neighbours if they shared boundaries, and the False Discovery Rate (FDR) correction was applied to account for multiple tests.

Then, to explore the landscape features that may be associated with a higher risk, three different models analysing risk index and risk components were built. The dependent variables were the population exposure index (model 1), the tick hazard index (model 2) and the risk index (model 3). The explanatory variables were fine-scale landscape features of the park, that includes both natural and man-made features. These include variables describing habitat structure and composition, infrastructure and/or attractions in the park and topography (Table 1), and the year of sampling. Variables describing the landscape characteristics and facilities around each trail segment (Table 1) were derived using using various GIS data sources as follows. Provincial digital forest cover maps [46] were used to calculate the proportion of forest cover around each trail segment. Then, a georeferenced layer of trails and park infrastructure obtained from the park authorities (unpublished data) was used to derive trail (trail density, including width and length in meters and the number of connections between the trails) and infrastructure (proximity of entrances, refuges, viewpoints, and service areas) metrics. Fragmentation metrics were then calculated using the forest cover map and the trail and park infrastructure layer, where trails and boundaries between two habitat types were considered as to fragment the forest into patches [25, 29]. The mean size of the patches adjacent to the trail segments and the edge density between the forest and another habitat type around the trail segments were calculated (as detailed in Table 1). Finally, the mean elevation in meters along the trail segments was extracted from a digital terrain model derived from LiDAR [47]. All variables were constructed with *ArcGIS Pro* version 2.8.0.

**Table 1:** Description of the variables tested in the tick hazard, population exposure and risk of tick-human contacts models

Variable	Units	Expected relationship with DIN (tick hazard)	Expected relationship with visitor density (population exposure)
Forest cover	Proportion covered by forest habitat around the trail segment (buffer sizes: 100, 200, 300, 400 & 500 m)	+	+
Forest patch size	m^2^ (the mean size of patches adjacent to the trail segment)	-	-
Edge density between the forest and another habitat type	m of edges/ m^2^ around the trail segment (buffer sizes: 100, 200, 300, 400 & 500 m)	+	NA
Trail density	Trail width & length in m. No. of connections to other trail segments	+	+
Proximity of park entrances	Distance to or identification of the nearest entrance	NA	+
Proximity of park facilities and attractions	Distance to or identification of the nearest refuge, viewpoint or service area. Presence of a lake along the trail	+	+
Elevation	Mean elevation along the segment, in m	-	+

The statistical analysis were carried out in R version 4.1.3, and the model selection was performed as follows. First, all variable distributions were checked and normalized by applying log transformations when necessary. Then the effects of each variable were explored graphically and in univariate models using *p* < 0.25 as the criteria for variable retention. The presence of collinearity between the retained explanatory variables was verified, using a variance inflation factor (VIF) threshold of 3 [48]. Using the resulting set of covariables, linear models were fitted, in which optimal model structures were selected by backwards stepwise elimination. Residual spatial autocorrelation was tested using Moran’s I and correlograms, performed with packages *spdep* version 1.1.11 [49] and function *icorrelogram* [50]. Because all models showed strong spatial autocorrelation in their residuals, spatial trend surface models were then fitted. This type of model is used to deal with dependencies arising from environmental gradients, while allowing flexibility in capturing non-linear responses across geographic space [50]. The coordinates of trails segments centroid were used as covariates in these models, to which smoothing functions were fitted using package *mgcv* version 1.8.38 [51]. Lastly, the significance of explanatory variables and the presence of spatial autocorrelation in the model residuals was reassessed, and the fit of final models was verified using graphs and by using the diagnostics function gam.check of package mgcv version 1.8.38 [51].

## Results

### Tick hazard

**Fig. 2:**
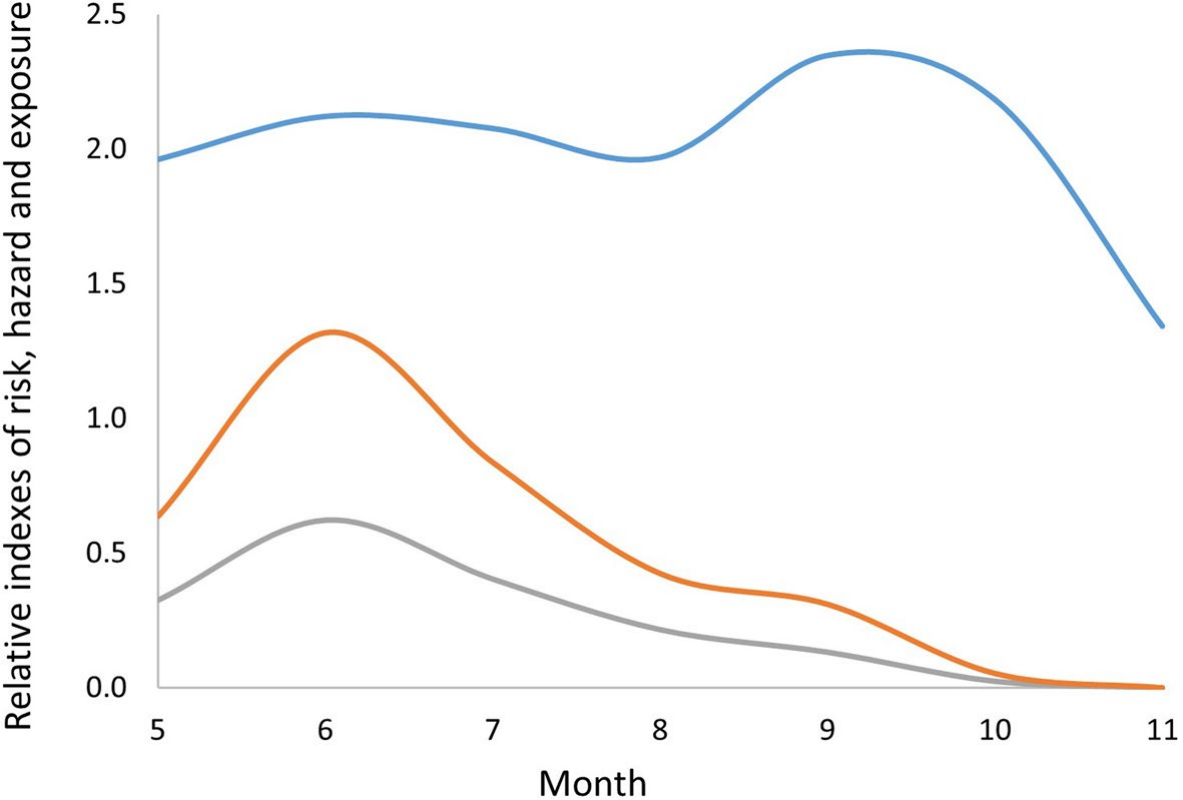
Average seasonal distribution of the risk index and its components, tick hazard (DIN) and exposure (no. of days-visits). A loess function was used on the averages of the monthly DIN (no. of infected nymphs/100 m^2^) data and the number of park visitors in both years. The number of visitors to the park is displayed after being divided by 100,000, to allow for better graphic visualization. The average risk index over the months was calculated by multiplying the two previous variables and represents a relative probability of human-tick contacts in the population visiting the park over this period

**Fig. 3:**
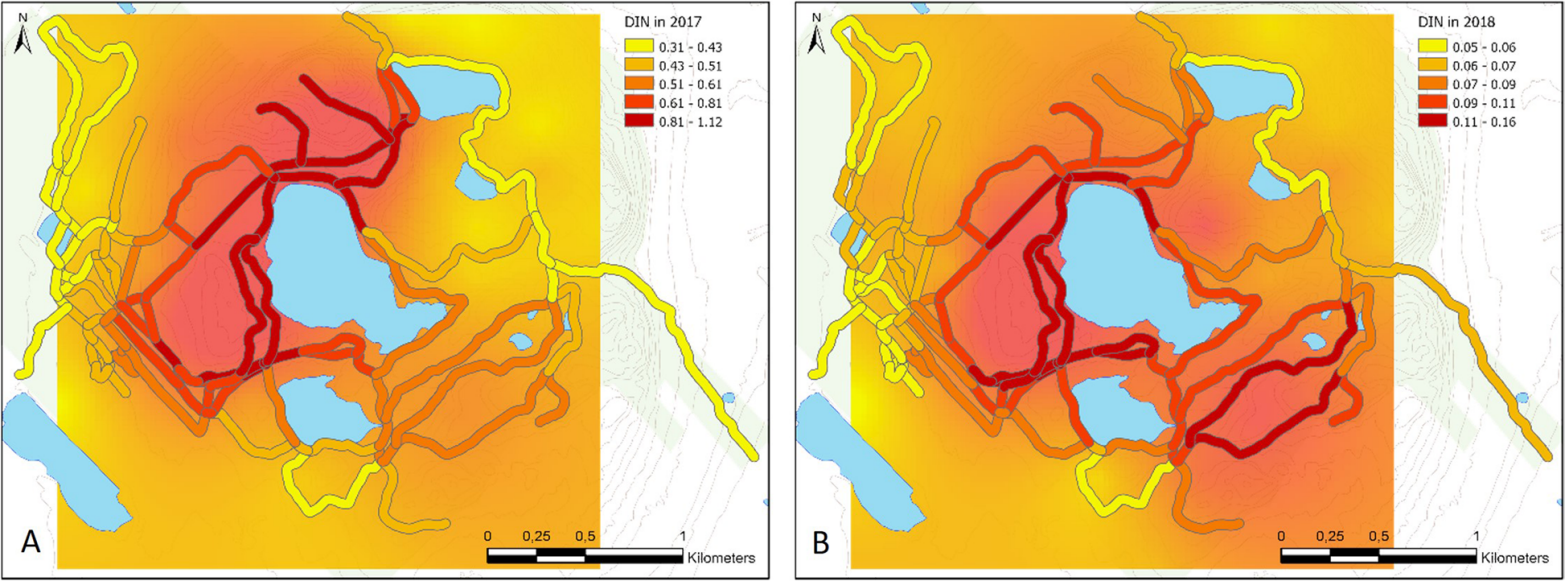
Spatial distribution of the entomological hazard index (density of infected *I. scapularis* nymphs; DIN) in the study site in 2017 **A** and 2018 **B**. Tick data were collected at sites located in the forest and interpolated with ordinary kriging models, then average values were extracted along each trail segment and its immediate surroundings (25 m buffer) and classified by quantile breaks, with classification based on the minimum and maximum values of each individual year (notably, 2017 exhibiting higher values than 2018)

All collected ticks in this study (*n* = 24,838) were identified as *I. scapularis*. On average, we found 19.3 larvae/100 m^2^ (range 0 to 263.8, standard deviation: ± 33.0), 3.2 nymphs /100 m^2^ (range: 0 to 42.7, standard deviation: ± 4.8) and 0.2 adults /100 m^2^ (range: 0 to 2.7, standard deviation: ± 0.4) per site-visit (more details on tick density can be found in Dumas et al. 2022). We found nymphs from May to October in both years, and a peak in density occurring in June (Fig. 2). We detected *B. burgdorferi* in 9.5% of nymphs tested (149/1542, ticks tested represented 49% of those collected (*n* = 3173)). The prevalence of infection in nymphs at sites ranged from 0 to 29.4%, with an average of 10.5% in 2017 (CI 95% [8.67%-12.68%]; *n* = 948) and 7.4% in 2018 (CI 95% [5.34%-9.95%]; *n* = 540). The density of infected nymphs collected at sampling sites ranged from 0.01 to 1.77/ 100 m^2^, with an average of 0.56/ 100 m^2^ in 2017 and 0.08/ 100 m^2^ in 2018 (Fig. 3). Standard error maps for the kriging interpolations are presented in the supplementary materials (Figure S1).

### Population exposure

With the trail cameras deployed in 2017, we collected 19,639 pictures over 194 sampling days. In these pictures, 82.10% displayed people on foot or bike, 15.90% were misfires, 7.13% displayed wildlife, and 1.29% were vehicles. The majority (91.35%) of the people detected were adults. The average group size of visitors was 1.53 (range: 1–15).

**Fig. 4:**
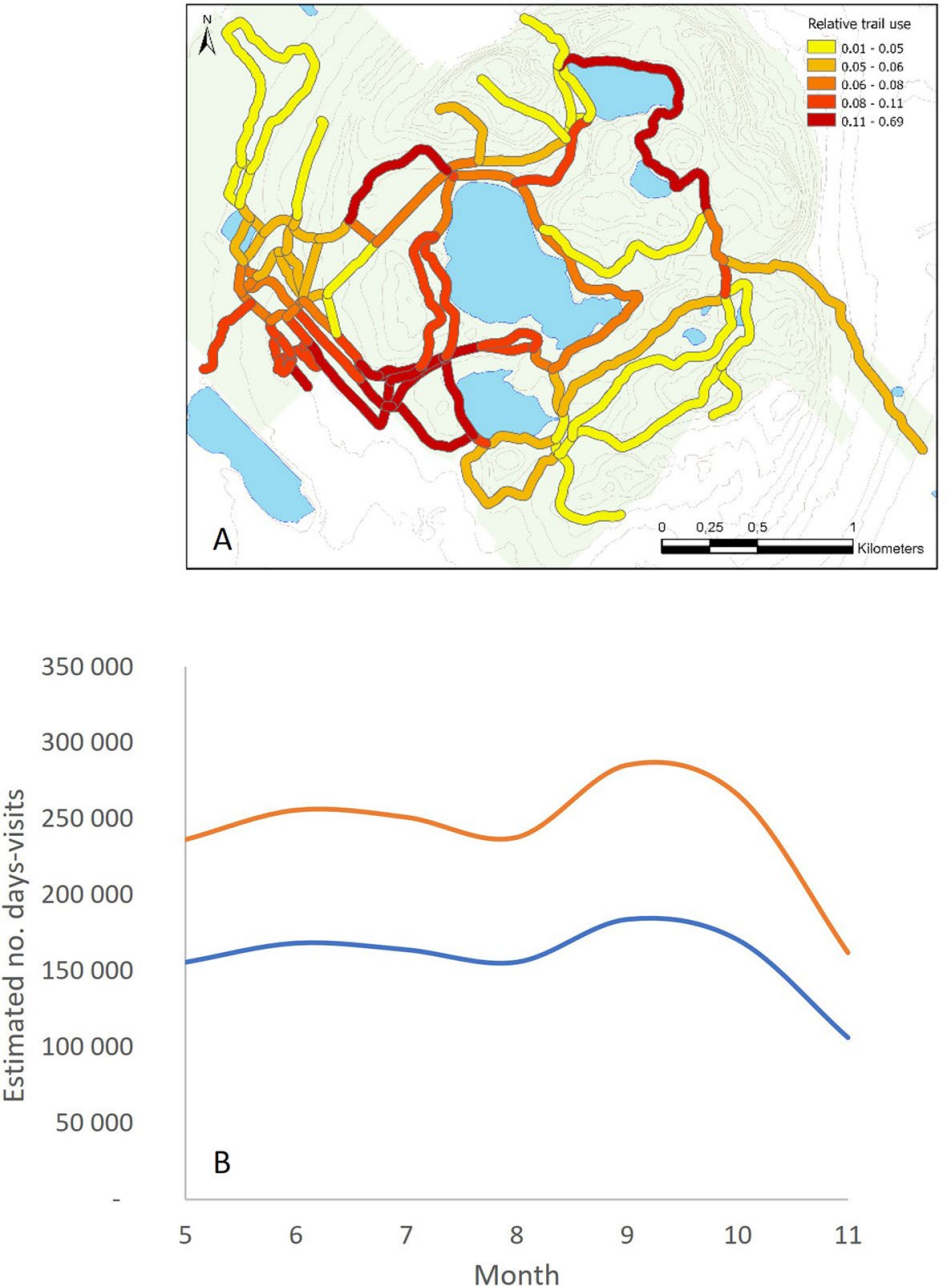
Spatio-temporal distribution of park attendance. **A** Relative trail uses as measured by counters (scaled from 0.01 to 1), interpolated over all trails in the park and classified by quantile breaks. **B** Variation in park use (number of days-visits) throughout the tick activity season in 2017 (blue curve) and 2018 (orange curve), obtained from the number of daily admissions recorded and the estimated number of visits by annual ticket holders

Park visitation varied among seasons and between the two years of the study (Fig. 4). There were two peaks in visitation, with the largest peak occurring in early fall (September to October) and a much smaller one in June to July.

The distribution of visitors across the park was heterogeneous, with areas located near the southwestern entrances to the park and trails surrounding lakes most heavily used (Fig. 4).

### Spatial and temporal distribution of risk

**Fig. 5:**
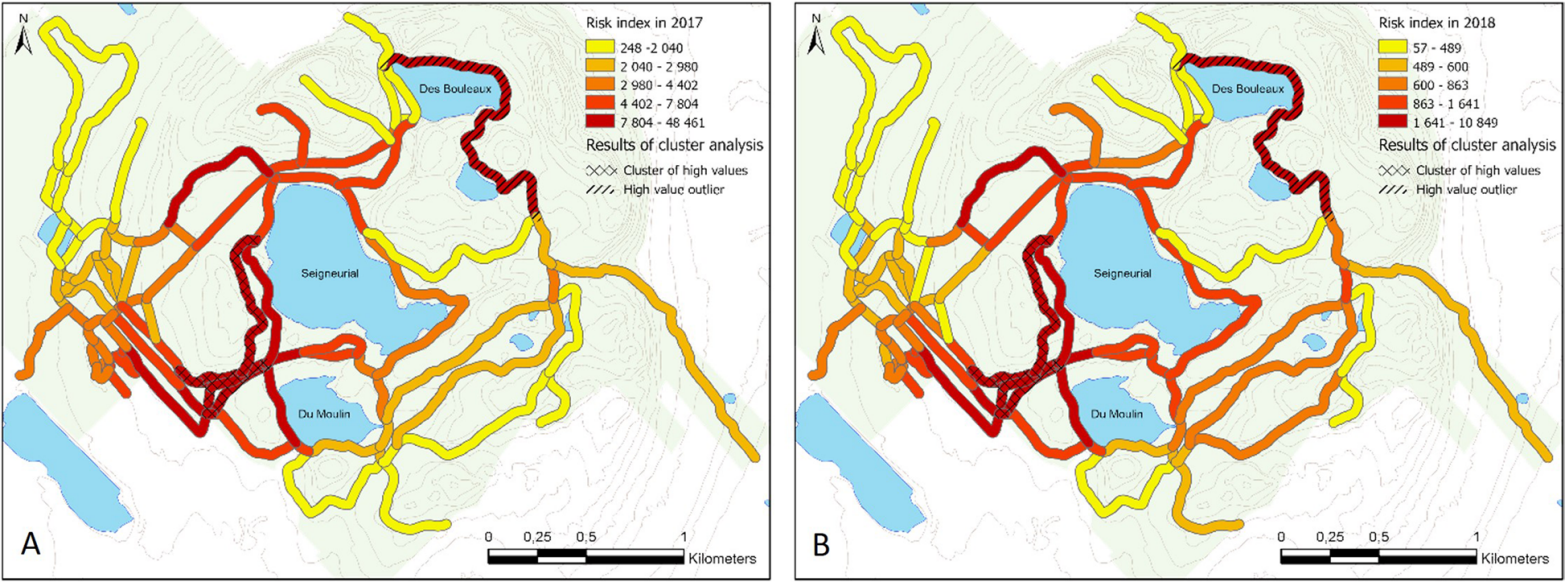
Spatial distribution of risk levels classified by quantile breaks and high-risk areas based on cluster analyses for 2017 (**A**) and 2018 (**B**). The risk index represents the probability of human-tick contacts in the population visiting the park

There was no significant correlation between DIN levels and trail use across the park (Pearson's correlation coefficient = -0.09, *p* = 0.20). However, there were specific areas where both indicators were high, resulting in spatial clusters of high risk of tick-human contacts. In both years, high-risk clusters were present around the trails connecting entrance 3 and Lake Seigneurial (Fig. 5). Also, the trail north of Lake Des Bouleaux had significantly higher risk index values than the surrounding trails. The rest of the areas did not show significant differences in their risk levels, except for a few areas with overall lower risk values (low-risk clusters, not shown). Significant high-risk areas (including high-risk clusters and outliers) encompassed 41% of the calculated 2017 risk index and 43% of the 2018 risk index, and in both cases covered 11% of the study area. Similar patterns were present in both years of the study, with some variation due to the varying spatial distribution of nymphs in the park in both years. The risk level was highest in June, when nymphs are also at their peak of abundance. A second, lower, peak of risk was present in September, when nymphs are less abundant, but more people used the park (Fig. 2).

Using the population exposure model, we found a positive relationship between the proportion of the area covered by forest within 100 m of the trails and the number of visitors (Table 2). We found the opposite relationship with elevation, where trails at higher elevations attracted fewer people than trails lower on the mountain. The number of visitors on trails also varied with distance from the three types of facilities in the park. Trails near the entrance 1 received 1.43 times more [95% CI: 1.15–1.71] visitors than those near the entrance 2, those near the refuge 4 received 2.23 times more [95% CI: 1.72–2.73] visitors than those near refuges 1 or 2, and those near the viewpoint 1 or 4 received 2.98 times more [95% CI: 2.31–3.65] visitors than those near the viewpoint 2. Differences between the other levels for these variables were not significant.

**Table 2:** Parameter estimates for the best spatial trend surface models of population exposure (model 1), tick hazard (model 2) and risk (model 3) indices, in 2017 and 2018. The risk index represents the probability of human-ticks contact in the population visiting the park

Parameters	β	SE	Edf	*P*
	**Model 1: Population exposure (density)** ^a^			
(Intercept)	9.01	0.16		< 0.001
Elevation	-0.35	0.15		0.03
Forest proportion (100 m radius)	0.28	0.05		< 0.001
Nearest park entrance				
* 2 vs 1*	-0.36	0.14		0.01
* 3 and 4 vs 1*	-0.17	0.27		0.53
* 5 vs 1*	-0.09	0.31		0.77
* 6 and 7 vs 1*	-0.92	0.57		0.10
* 8 and 9 vs 1*	-0.32	0.34		0.35
Nearest refuge				
* 2 vs 1*	-0.02	0.11		0.88
* 3 vs 1*	0.11	0.15		0.50
* 4 vs 1*	0.80	0.26		0.002
Nearest viewpoint				
* 2 vs 1*	-1.09	0.34		0.002
* 3 vs 1*	-0.46	0.25		0.071
* 4 vs 1*	-0.04	0.10		0.679
* 5 vs 1*	-0.10	0.20		0.612
Year				
* 2018 vs 2017*	0.43	0.03		< 0.001
Smoothed coordinates of trail segment centroids			27.2	< 0.001
	**Model 2: Tick hazard (DIN)** ^b^			
(Intercept)	4.06	0.01		< 0.001
Forest proportion (200 m radius)	0.04	0.02		0.026
Year				
* 2018 vs 2017*	-1.99	0.02		< 0.001
Smoothed coordinates of trail segment centroids			21.42	< 0.001
	Model 3: Risk (hazard*exposure) ^a^			
(Intercept)	8.24	0.10		< 0.001
Forest proportion (100 m radius)	0.24	0.05		< 0.001
Nearest refuge				
* 2 vs 1*	0.01	0.12		0.95
* 3 vs 1*	0.04	0.16		0.80
* 4 vs 1*	0.84	0.27		0.002
Nearest viewpoint				
* 2 vs 1*	-0.76	0.34		0.03
* 3 vs 1*	-0.53	0.29		0.07
* 4 vs 1*	-0.01	0.11		0.90
* 5 vs 1*	-0.28	0.20		0.17
Year				
* 2018 vs 2017*	-1.56	0.03		< 0.001
Smoothed coordinates of trail segment centroids			27.61	< 0.001

^a^Logged variables

^b^Multiplied by 100 and logged variable

Of the predictors tested in the tick hazard model, only the proportion of the area covered by forest within 200 m of the trail was positively associated with a greater density of infected nymphs (Table 2).

In the risk model, the proportion of forest within 100 m of the trail was also associated with higher risk levels. Risk levels also varied with proximity to park facilities (Table 2). Trails near refuge 4 had 2.32 times the risk level [95% CI: 1.80–2.85] as those near the refuges 1 or 2, while those near the viewpoint 1 had 2.13 times the risk level [95% CI: 1.47–2.80] as those near the viewpoint 2. Differences between the other levels for these variables were not significant.

Population density (exposure) was 1.53 times higher in 2018 than in 2017 [95% CI: 1.48–1.59]. On the other hand, the tick hazard was 7.31 times higher in 2017 than in 2018 [95% CI: 1.48–1.59] and the resulting risk level was 4.76 times higher in 2017 than in 2018 [95% CI: 4.70–4.83]. The final population density, tick hazard, and risk models explained 94.4%, 98.9%, and 96.5% of the variation in deviance, respectively, and the root mean square errors for these model predictions relative to observations were 0.20, 0.11, and 0.23, respectively. Predictions from the three models and variation statistics for tested covariates are presented in the supplementary materials (Figure S2 and S3, Table S1).

## Discussion

While many studies have focused on factors driving tick-borne disease incidence at large geographical scales in North America, very few have investigated simultaneously the ecological and human population factors that may determine risk at finer scales. It is however important to better understand the factors at play at this scale, because it is at this scale that interventions to prevent infections occur. In this study, we demonstrate the applicability and utility of an integrative risk assessment approach to estimate the probability of contacts between visitors and infected ticks, at a local intervention scale in a periurban park environment. The methodology employed allowed for the identification of high-risk areas and periods in park, demonstrating its utility as a planning tool in risk mitigation intervention plans in the context of natural public parks. In addition, we identified biophysical attributes that were associated with the risk levels across a highly visited and newly LD-endemic park. The relationships uncovered between fine scale landscape attributes and spatial variability in risk provide key findings on the ecological determinants of tick-borne disease in recreational parks, which may be a significant source of exposure to tick-borne pathogens for populations in urban areas.

First, we showed that the proportion of forest cover around trails was associated with higher levels of risk and influenced both the visitor density (exposure) and the density of infected nymphs (hazard). These results align with previous research indicating that forested habitats are associated with the population exposure to *B. burgdorferi*-infected ticks and high population incidence rates of Lyme disease cases compared to other habitat types (e.g., herbaceous and shrubby environments, agricultural areas, urban areas, or wetlands; [16, 52]. Trails located in areas where forest cover was dominant were more popular, consistent with the hypothesis that visitors may be more attracted to undisturbed forest areas in parks [53]. Also, infected nymphs were more abundant in areas where forest cover was dominant. This result is consistent with previous observations associating *I. scapularis* density with proportion of forest cover at several geographic scales [54]. However, in contrast to what was found in other studies performed at regional scales, we did not find a relationship between the DIN and forest fragmentation indicators or high human presence [55–58]. At smaller spatial scales, some studies performed in parks found lower DON or DIN levels in park areas with higher public use [30, 33]. In contrast, here, we did not observe a correlation between estimated DIN and trail use levels. We also found no relationship between DIN and indicators of the territory's accessibility such as distance to entrances, or indicators of forest habitat fragmentation such as edge and trail density or the size of the forest patches. It has been suggested that human disturbance of habitat may affect host presence and tick survival, and thus, that the lowest tick densities would be found in forested areas with the greatest current or past human presence [30, 33]. The opposite thesis has also been put forward, that the main hosts of blacklegged ticks (white-footed mice, white-tailed deer) adapt well to disturbed habitats and thus may become dominant there at the expense of other wildlife species, which are more sensitive to habitat disturbances [22]. Thus, since ticks would have more opportunity to encounter reproductive and reservoir hosts, their survival and reproduction, as well as the circulation of tick-borne pathogens, would be favored, resulting in a higher level of tick hazard [22]. It appears that in our context, neither of these hypotheses apply. Overall, there is relatively low mammal biodiversity across the park, with mice and deer present and abundant in the majority of areas [59]. The lack of relationship between DIN and fragmentation indicators therefore suggests that the main hosts, mice and deer, are not affected by fragmentation at this fine scale, and are present at sufficient abundances in a range of habitats throughout the park [59]. However, we did observe a positive relationship between the proportion of forest cover and the DIN. This suggests that in this context, it is the presence of a habitat favorable to the survival of ticks when they are off-host, i.e. a forest floor where the abiotic conditions (temperature, relative humidity, presence of refuges under the leaf litter) necessary for their survival are present, that is the main determinant of their distribution here [59].

Based on these results, we are suggesting approaches to manage the risk associated with ticks in natural parks. We recommend that actions aimed at decreasing the likelihood of human-tick contact be taken in areas of parks where forest cover dominates. For example, more emphasis should be placed on encouraging the adoption of safe behaviors by users, particularly in forested areas of parks. These best practices include staying on trails, using tick repellents, wearing long clothing, and practicing tick checks after a forest activity [20, 60]. These practices could be reminded to visitors directly in the parks, through signage in high-risk forest areas, to reinforce their adoption levels. Second, we recommend that trail edges in high-risk areas be landscaped so that the likelihood of human-tick contact is restricted. Trail maintenance that discourages contact includes regular trimming of vegetation along trails and removal of dead leaves from the ground [61] and installation of wood chips on the ground along trail borders [62]. Finally, reducing the probability of human-ticks contact can also be achieved by reducing the tick hazard in high-risk habitats. For this, possible interventions include the selective use of acaricides applied to vegetation in high-risk areas and host-targeted interventions (e.g., treatments with acaricides [63, 64]). However, all these interventions are resource-intensive, which currently limits their deployment over large areas such as the territory of natural parks [65]. Therefore, we propose here to deploy them first in high-risk areas to optimize the cost–benefit of deployment. Our cluster analyses have shown the presence of risk hotspots. By strategically prioritizing interventions to these hotspots, we could act on 41 to 43% (depending on the year) of the risk in the entire park, while deploying resources to only 11% of the territory. Such hotspots, when present in a park, are therefore places where high impact potential is possible if interventions are deployed. Slight variations in the location of high-risk areas were however present in this study, consistent with previous findings of heterogeneous patterns of tick densities at small spatial scales [30, 59, 66]. Therefore, periodic reassessment of the location of high tick density areas should be included in park risk management plans. This would ensure that interventions are always deployed in areas where their impact is expected to be the highest.

Second, we showed that proximity to certain facilities was associated with elevated risk. Specifically, we found certain park features (refuges, viewpoints and entrances) associated with increased levels of exposure and risk. This result is consistent with other studies that have found these types of elements to be associated with the level of attractiveness or accessibility of public natural areas [26, 28, 53]. This finding offers another opportunity for risk management, that of targeting population exposure patterns. Indeed, parks could be designed so that a mismatch between the location of areas of high population exposure and high tick hazard is induced. This could be accomplished by modifying the attractiveness and accessibility of areas, which are important drivers of visitor distribution across territories [19, 67]. As part of this approach, parks could develop trails, refuges, and lookouts in areas with low tick densities, so that people will tend to use these areas more. They could also limit public access to areas with high tick densities, especially during high-risk periods.

The main risk period in this study corresponded with the peak in nymph abundance [68, 69]. A second, lower peak in risk was present in early fall, when entomological risk is lower, but when the park receives large number of visitors. We did not, however consider a possible increase in entomological hazard associated with the presence of adult ticks in the fall and thus, the second peak could be underestimated by our analysis. In addition, tick densities were lower in 2018 than in 2017. While it is theoretically possible that tick removal by our sampling efforts could have influenced these results, it appears more likely that this variation is caused by weather fluctuations during these years [70], likely due to high summer temperatures and reduced rainfall in 2018. Indeed, our results are aligned with trends observed in active surveillance which showed higher generalized tick densities in 2017 than in 2018 across the province [71, 72].

Here, we conceptualized different levels of risk based on the presence and abundance of pathogen-infected vectors and the human population at risk, in the same space–time. As illustrated above, the approach developed here could be used as a decision support tool for risk management in the context of public parks. However, we cannot conclude on the link between the observed patterns and the actual incidence of LD in the local population. Several other factors could influence the actual patterns of disease acquisition by the local population, such as individual behaviors (i.e., what people are doing in the natural space and for how long), levels of knowledge and perception of risk in relation to the disease [7, 20], use of tick bite prevention methods or availability of medical management following a tick bite [73]. In particular, we could not consider how user behavior might influence their level of exposure to ticks in the environment. For example, we could assume that the actual exposure of people using the wide trails is less than for the narrow trails. In the former case, users may come into contact with the vegetation surrounding the trail or leaf litter at the edge of the trail less frequently than when using narrow trails. In the former case, users may come into contact with the vegetation surrounding the trail or leaf litter at the edge of the trail less frequently than when using narrow trails. Since trail edges can be favorable micro-habitats for ticks [30], different exposure to them could impact users' actual exposure to ticks. In addition, bicycle use was only allowed on the wide trails in the park. If it also turned out that cyclists were less exposed to tick bites than pedestrians, then our estimate of the level of risk on the wide trails could be underestimated. On the other hand, it is also possible that our analysis underestimates risk in some areas of the park. For example, if certain attributes attracted people to go off trails, or to pick things off the ground, these areas could be a source of greater exposure to ticks. Such observations were made in a French park, where areas conducive to picking plants and mushrooms represented the highest risk of exposure to *Ixodes ricinus* ticks for users [74]. Further studies would be needed to assess the effect of users' behaviors on their individual risk of exposure to ticks. Another limitation of this study is the precision of the pathogen prevalence estimates used in the calculation of the tick hazard indices. Ideally, these estimates should be calculated at the site level, but due to limited sample sizes and testing capacity, site-level estimates were too uncertain. Instead, we opted to use park-level estimates, providing higher confidence. While this approach is supported by previous studies showing that the main driver of local DIN is generally DON [75, 76], larger sample sizes in future studies would increase confidence in local risk assessments. Finally, the risk levels obtained here at the scale of the park would also need to be validated with accurate disease acquisition data. However, the level of detail that would be required on such a fine scale is difficult to obtain in the context of vector-borne diseases. Indeed, the locations of acquisition of infection by vector-borne diseases are often unknown or confidential. Modeling studies could then be used to simulate the dynamics of encounters between the populations described here and the subsequent steps that could lead to the development of the disease in bitten individuals, for example. The proposed strategy of prioritizing intervention in high-risk areas should also be tested in different epidemiological scenarios to demonstrate its effectiveness.

## Conclusion

This study demonstrated that the risk of contact between visitors and *Borrelia burgdorferi*-infected ticks within a public park of southern Quebec, Canada, varied with landscape features and infrastructure at a fine geographic scale. We found a positive relationship between forest cover and the risk index, but no other associations could be found regarding other indicators of fine-scale forest fragmentation. This raised questions about the inter-scale generalizability of previous findings − the large majority of which were made at coarser spatial resolutions − about the links between forest fragmentation and tick-borne disease risk in various human-altered ecosystems. Thus, we believe that continued research efforts investigating these relationships remain important and should incorporate more fine-scale data on the distribution of ticks and people in at-risk habitats. Finally, we also demonstrated a practical methodology for integrative risk assessment that can be used to guide risk management efforts by local stakeholders and translated to other contexts as part of One Health approaches to emerging infectious diseases management.

### Supplementary Information

**Supplementary Material 1.**

## Data Availability

The datasets used and/or analysed during the current study are available from the corresponding author on reasonable request.

## Abbreviation

LD: Lyme disease
DIN: Density of infected nymphs
DON: Density of nymphs
NIP: Nymphal infection prevalence
