# Supplementary material for: The risk of contact between visitors and Borrelia burgdorferi-infected ticks is associated with fine-scale landscape features in a southeastern Canadian nature park
Source: BMC Public Health. 2024 Apr 26;24:1180. doi: 10.1186/s12889-024-18673-w (PMC11055428; doi:10.1186/s12889-024-18673-w)
Supplement: Supplementary file 1 — Supplementary Material 1. [file 12889_2024_18673_MOESM1_ESM.docx]

*BMC Public Health*

**Supplemental material**

**The risk of contact between visitors and *Borrelia burgdorferi*-infected ticks is associated with fine-scale landscape features in a southeastern Canadian nature park**

Ariane Dumas, Catherine Bouchard, Pierre Drapeau, L. Robbin Lindsay, Nicholas H. Ogden and Patrick A. Leighton.

**Table of contents**

**Figure S1:** Standard error of prediction for ordinary kriging interpolation between tick sampling sites.

**Figure S2.** Population exposure, tick hazard and risk levels in Mont Saint-Bruno National Park, predicted by GAM trend surface models (from low values in yellow to high values in red and classified by 5 quantile breaks^1^). Circles positioned at the centroids of each trace segment represent the residuals of the predictions, in red for negative values and green for positive values. The size of the circles is proportional to the absolute value of the residuals^2^

**Figure S3.** Comparison of predictions with observations for model 1, population exposure (A), model 2, tick hazard (B), and model 3, risk (C). The uncertainty of the predictions is illustrated by the confidence intervals (CI 95%) which are represented by the dashed red lines.

**Table S1**: Variation statistics of fine-scale landscape features, selected as numerical variables for model building following initial data exploration. Prior to final modeling, these variables were centered and scaled.

Figure S1: Standard error of prediction for ordinary kriging interpolation between tick sampling sites (black dots).


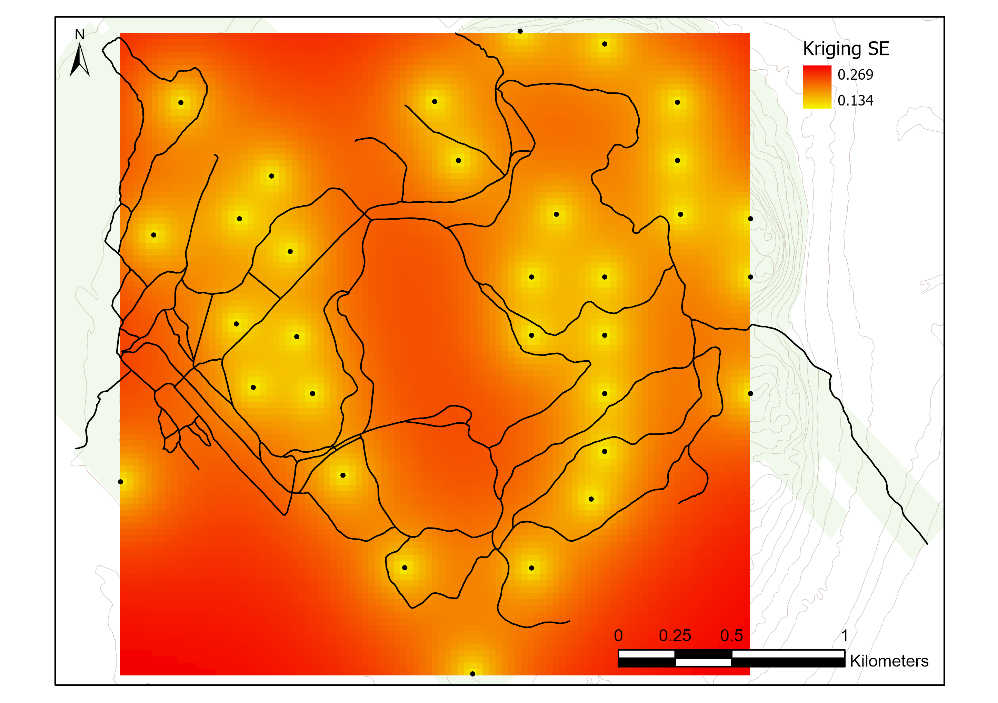


Figure S2. Population exposure, tick hazard and risk levels in Mont Saint-Bruno National Park, predicted by GAM trend surface models (from low values in yellow to high values in red and classified by 5 quantile breaks^1^). Circles positioned at the centroids of each trace segment represent the residuals of the predictions, in red for negative values and green for positive values. The size of the circles is proportional to the absolute value of the residuals^2^.

| **2017** | **2018** |
| --- | --- |
| **Model 1: Population exposure** | |
| 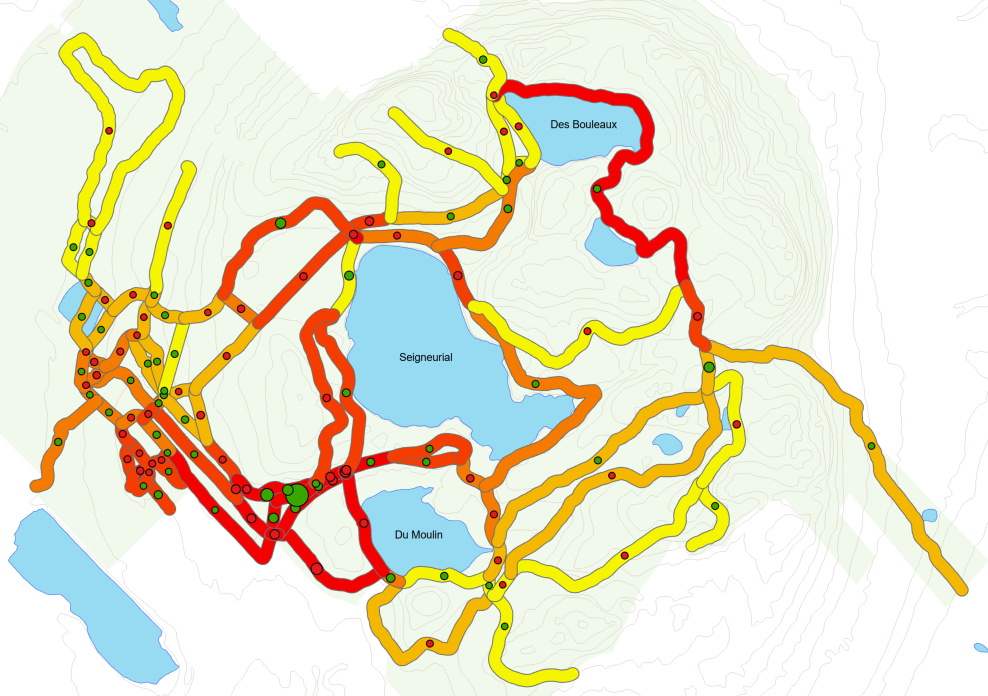 | 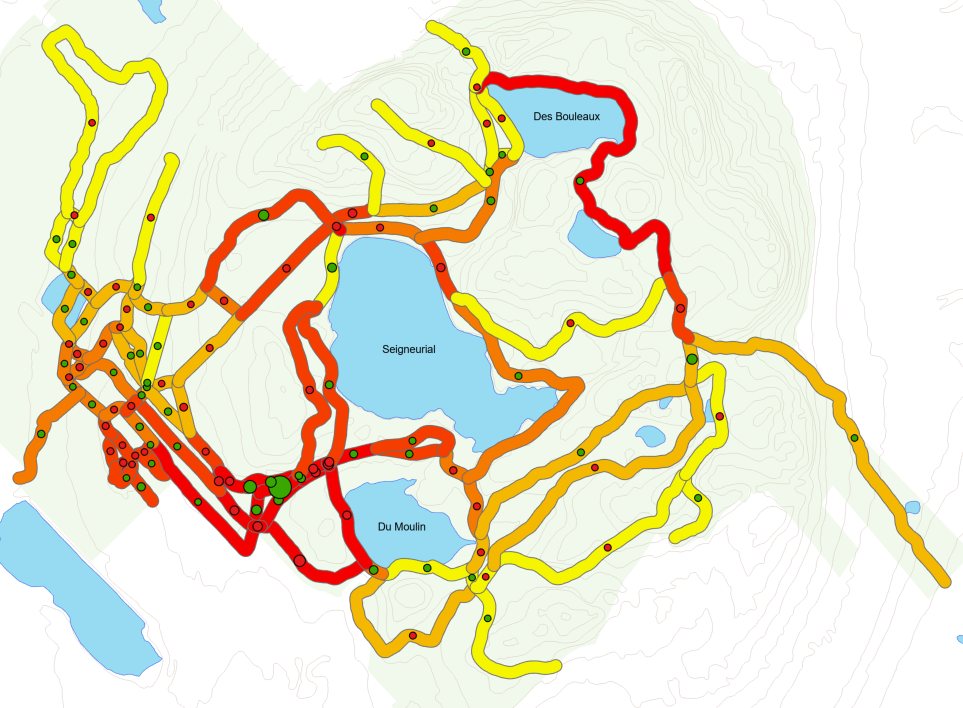 |
| ^1^Predictions range: 6.53 ̶ 10.60  ^2^Residuals range: -0.76 ̶ 0.71 | ^1^Predictions range: 6.96 ̶ 11.03  ^2^Residuals range: -0.76 ̶ 0.71 |
| **Model 2: Tick hazard** | |
| 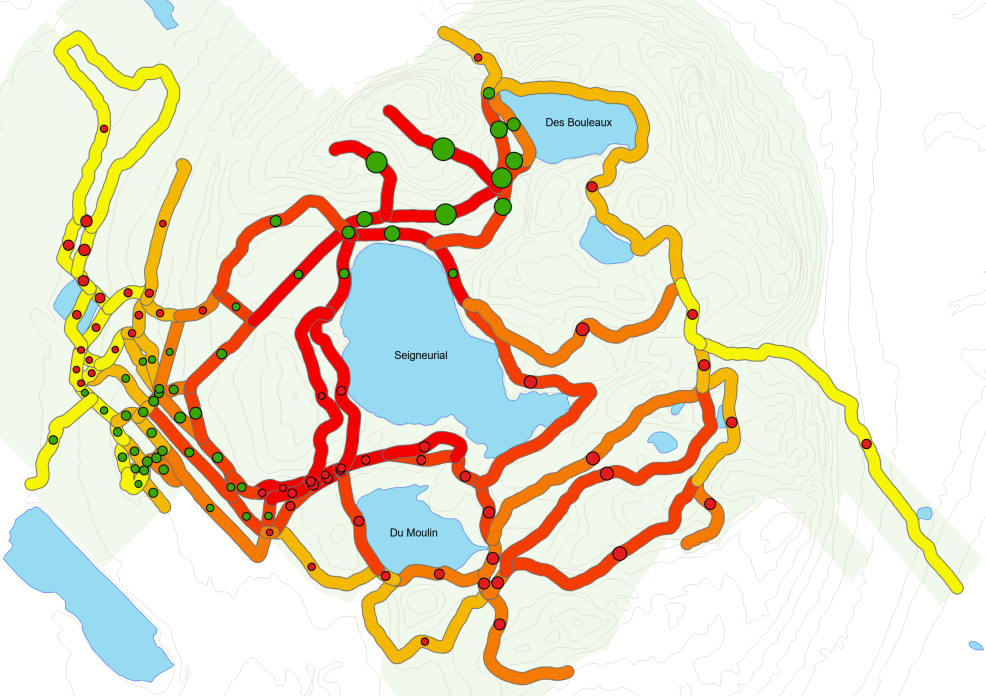 | 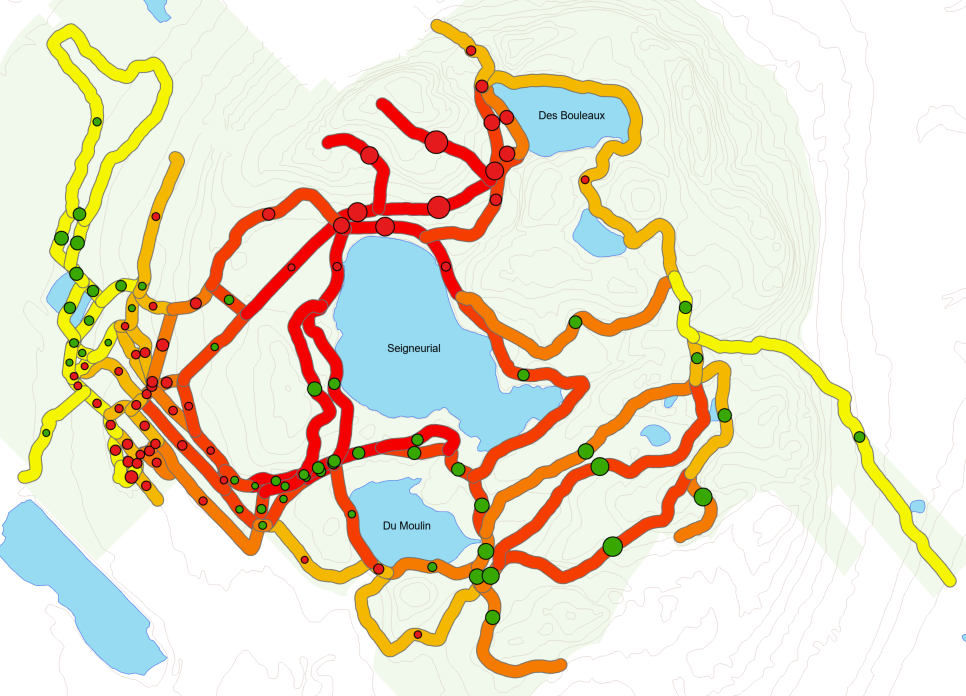 |
| ^1^Predictions range: 3.62 ̶ 4.66  ^2^Residuals range: -0.22 ̶ 0.28 | ^1^Predictions range: 1.63 ̶ 2.67  ^2^Residuals range: -0.28 ̶ 0.21 |
| **Model 3: Risk** | |
| 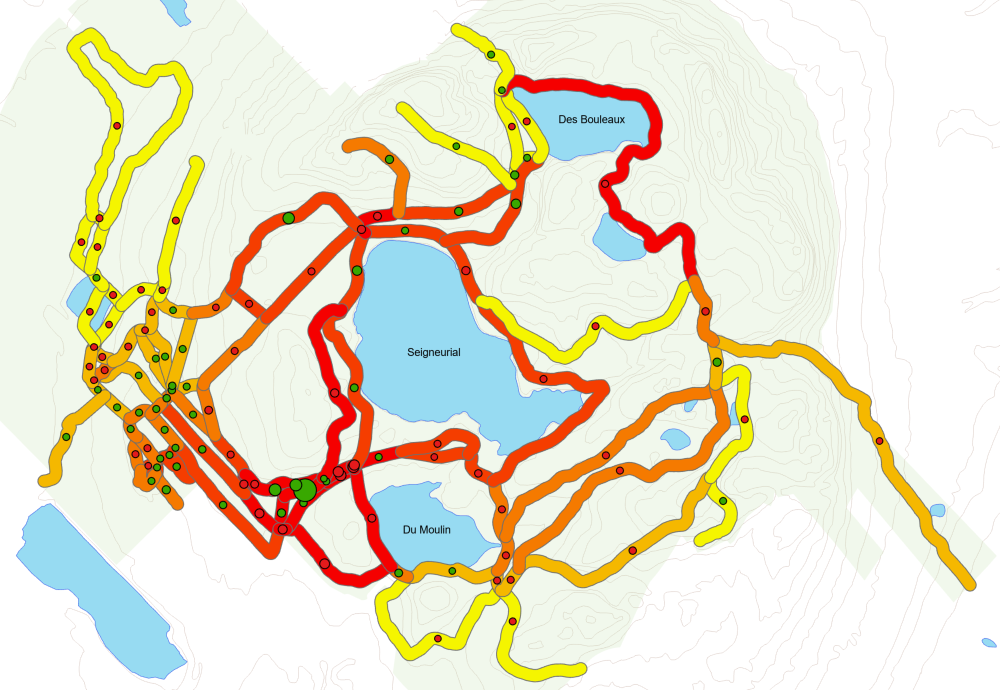 | 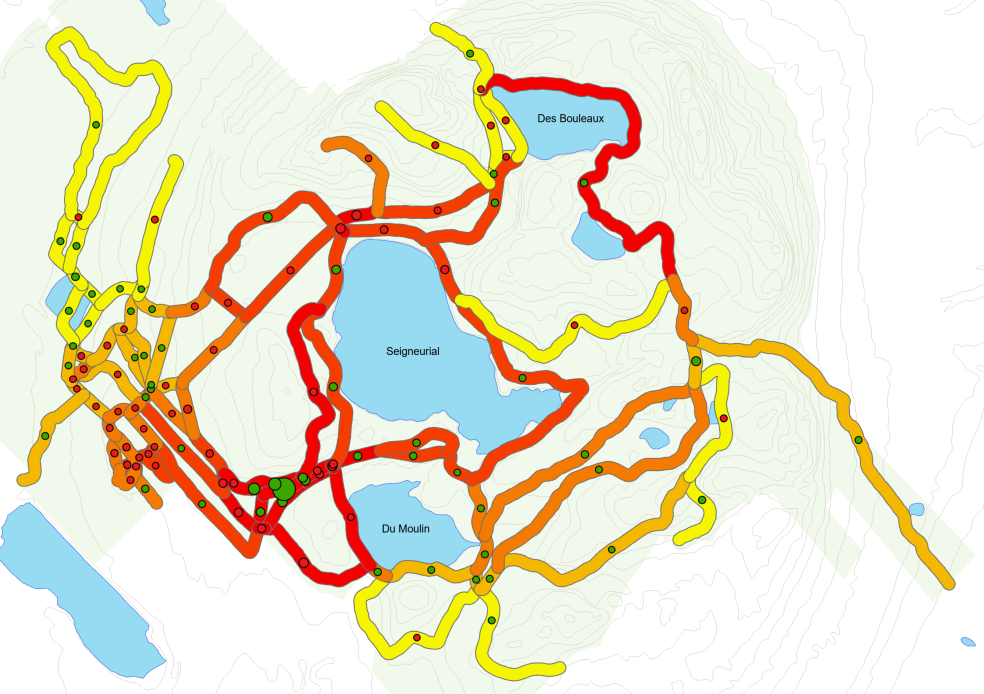 |
| ^1^Predictions range: 5.57 ̶ 10.37  ^2^Residuals range: -0.90 ̶ 0.76 | ^1^Predictions range: 4.01 ̶ 8.81  ^2^Residuals range: -0.72 ̶ 0.82 |

Figure S3: Comparison of predictions with observations for model 1, population exposure (A), model 2, tick hazard (B), and model 3, risk (C). The uncertainty of the predictions is illustrated by the confidence intervals (CI 95%) which are represented by the dashed red lines.


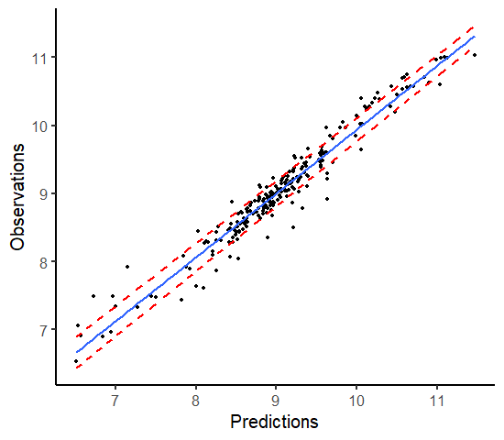

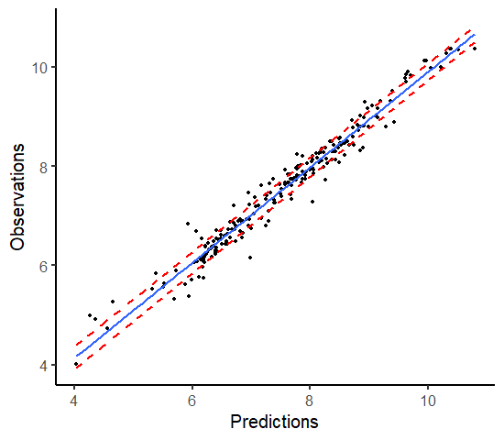

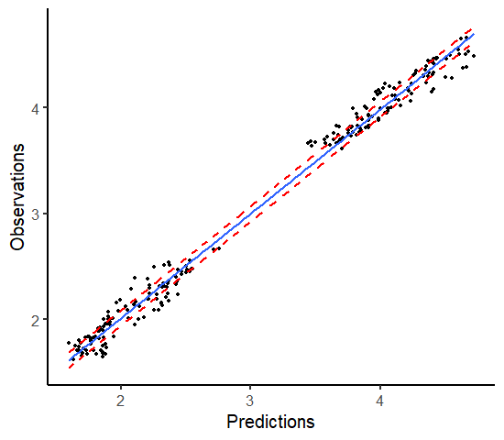


A

B

C

Table S1: Variation statistics of fine-scale landscape features, selected as numerical variables for model building following initial data exploration. Prior to final modeling, these variables were centered and scaled.

| **Variable** | **Mean** | **SD** |
| --- | --- | --- |
| trail_width | 2.768 | 0.975 |
| trail_segment_length_log | 318.278 | 376.514 |
| mean_elevation | 89.865 | 24.560 |
| distance_nearest_entrance | 347.301 | 237.201 |
| no_connections | 4.125 | 0.759 |
| forest_patch_size_log | 11.311 | 2.190 |
| forest_cover100m | 0.693 | 0.272 |
| forest_cover200m | 0.675 | 0.213 |
| forest_cover300m | 0.651 | 0.178 |
| forest_cover400m | 0.638 | 0.152 |
| forest_cover500m | 0.622 | 0.133 |
| edges_density100m | 0.005 | 0.004 |
| edges_density200m | 0.005 | 0.002 |
| edges_density300m | 0.004 | 0.001 |
| edges_density400m | 0.004 | 0.001 |
| edges_density500m | 0.004 | 0.001 |
